# Laparoscopic drama

**DOI:** 10.1590/S1516-31802002000100009

**Published:** 2002-01-02

**Authors:** Carlos José Benatti

Various philosophers have drawn the attention of scholars of social phenomena to the issue of the gulf between image and substance. Feuerbach says that there is a preference in our times for sign over essence, copy over original, representation over reality, appearance over original, representation over reality, appearance over essence. Borstin argues that non-reality is the dominant factor in the construction of contemporary experiences and that there is a real fetish for what's new. Pseudo-events are created because of the shortage of real happenings and news, which used to bring tidings but today present old events misted over with superficial reports.

We live in a world in which cinema and TV now allow a prodigy: a world where image appears more true to life than the original, and where fantasy is more veridical than reality. Therefore, this whole issue is about the construction of a society based on image, in other words, the ‘showmanization’ of events to produce a spectacle society.

The laparoscopist always needs to be aware of these thoughts so as not to transform his noble craft into a simple spectacle, so that the surgeon does not create a "Homo spectator". A good test would be to ask oneself whether there has not been a evolutionary halt in one's laparoscopic activities, showing a certain rapture, a certain anesthetization towards images. A depiction of the world that transcends the real world. The laparoscopist cannot be transformed into a voyeur. The characteristic of a voyeur is to invade spaces, to spy, without acting, gaining great pleasure from the act of spying.

The history of laparoscopy has many parallels with the story of film, but in the former the camera has consolidated supremacy over the human eye and vision has consolidated supremacy over the other senses. In laparoscopy, we see the pus but can neither smell nor touch it. The world of the screen has consummated all our feelings and fantasies as well as laparoscopists’ projects. Cinema, like laparoscopy, is a contemporary myth with its rituals, its temples and its followers. Cinematographic vision, like laparoscopy, is hegemonic and omnipresent. Nothing escapes it. The metaphor of laparoscopy is the ‘showmanization’ of disease, just as the cinema's metaphor is a ‘showmanization’ of society.

In fact, laparoscopy while working with a single frontal lens reminds us of the frontal nature of theater, where one does not have various simultaneous angles on the phenomenon observed and close-ups, as is the case with cinema. Theater and cinema are illusionist arts, using many devices, whereas the discourse of laparoscopy is committed to reality. In the theater, one is present at the very moment events are taking place (on-line, to use modern language), the same as in laparoscopy. On the other hand, the link between images is true of both cinema and laparoscopy.

The metaphor of the theater is static reality, while dynamic reality is the metaphor of cinema and laparoscopy. All these are really dramatic metaphors, with the act (the surgery performed), the scene (surgical-stage), the agent (surgeon), the means (to change, etc.), and the purpose (indication of surgery).

All this goes to show that reality does not exist and is no more than a social construction. Cinema was born along with psychoanalysis at the beginning of the 20^th^ century, and took twenty years to develop color and sound, things that we laparoscopists did not have to wait for. As of this date, cinema still has many links with psychoanalysis whilst we laparoscopists, bastard sons of the cinema as we are, have never had any links with it. Or have we?

